# Model-based cost-effectiveness analysis of external beam radiation therapy for the treatment of localized prostate cancer: a systematic review

**DOI:** 10.1186/s12962-019-0178-3

**Published:** 2019-05-21

**Authors:** Solomon Kibret Abreha

**Affiliations:** University of Insubria, Varese, Italy

**Keywords:** Cost effectiveness, Markov model, Decision analysis, Radiation therapy, Economic evaluation, Prostate cancer

## Abstract

**Background:**

External beam radiotherapy is the recommended but expensive treatment option for localized prostate cancer. Prostate cancer is the most common cancer in men worldwide. A cost-effectiveness study is needed given the excessive cost of radiotherapy treatment and the high prevalence of prostate cancer. The aim of this systematic review was to assess and identify studies that examined model based economic evaluation of external beam radiation therapy for the treatment of localized prostate cancer.

**Methods:**

A systematic review of the published literature was conducted through MEDLINE, NHS EED (NHS Economic Evaluation Database), and Cochrane databases with a specific search strategy. The literatures were searched according to the preferred reporting items for systematic reviews and meta-analyses (PRISMA) statement. At first 1046 citations were identified. The extracted files were imported into the Rayyan systematic review site for inclusion or exclusion based on the defined criteria. Studies included in this review were articles published between 2003 and 2017, and that conducted full-economic evaluations of the modality of external beam radiotherapy for the treatment of localized prostate cancer.

**Results:**

There were 12 studies that satisfied the inclusion and exclusion criteria. Seven studies compared intensity modulated radiation therapy (IMRT) with three-dimensional conformal radiation therapy (3D-CRT), two compared IMRT with stereotactic body radiation therapy (SBRT) another two-paper assessed IMRT with proton beam therapy (PBT). One paper compared the three external-beam radio therapy options of IMRT, SBRT and PBT. Most of the studies were originated from the US and analyzed the cost data from the payer’s perspective. Most studies were supported that IMRT was cost effective when it compared with 3D-CRT. Compared with IMRT, SBRT was found to be cost-effective.

**Conclusions:**

There are limited number of studies exist on the cost effectiveness of radiation therapy options for the treatment of localize prostate cancer across Europe. Most studies are originated from the US Medicare payer Perspective. Further research is need that investigate the cost effectiveness of these radiation therapy options from the societal perspective in Europe.

**Electronic supplementary material:**

The online version of this article (10.1186/s12962-019-0178-3) contains supplementary material, which is available to authorized users.

## Background

There is an increasing use of economic evaluation in healthcare to inform medical decision making. Drummond et al. [1] defined economic evaluation in healthcare as “the comparison of alternative options in terms of their costs and consequences”. Economic evaluations are important in decisions about investment in modern technologies and for the rational choices between different treatment options. Cost-effectiveness studies of cancer in general and localized prostate cancer in particular is important because it helps patients, physicians, and policymakers make quantitatively-based decisions, which balance treatment efficacy, toxicity, and costs. Cost-effectiveness analysis allows for quantitative comparison of different treatments for the same condition and allows one to weigh the difference in costs between two or more treatment options against the difference in effectiveness. Cost-effectiveness research of localized prostate cancer treatments can have a significant impact on healthcare spending due to the high prevalence of prostate cancer and the many treatment options with similar outcomes, yet substantially different costs. Therefore, cost-effectiveness studies for localized prostate cancer are paramount as they may save the health care system millions of dollars while optimizing the value of care delivered to patients.

Prostate cancer is the most common cancer in men worldwide. It is the most common malignancy diseases in males in Europe and North America. The American Cancer Society estimated 164,690 new cases of prostate cancer, with 29,430 prostate cancer-related diseases in the United States by the year 2018 [2]. It is the most common neoplasm among men and third-ranked cause of cancer death in Europe, with almost 400,000 cases and over 92,000 deaths [3]. In England and Wales each year there are about 27,773 new cases and 9161 deaths [4]. More than half of the men diagnosed with prostate cancer in the UK each year are aged 70 and over. The economic impact of prostate cancer in an aging population is expected to be high due to the high prevalence of prostate cancer. In the UK the mean direct costs per patient for initial treatment for prostate cancer have been estimated at around £2505. This figure compares to £2572 in Spain, £3205 in Germany, £4129 in Italy, and £4622 in France [4]. The total estimated costs for all patients in the first year from diagnosis were estimated to be £94.1 million in the UK compared to £92.5, £196.9, £163.0 and £310.6 million in the other countries respectively [4].

Localized prostate cancer (stage 1 and 2) is the stage in which the cancer is only in the prostate and has not spread anywhere else in the body. Radiotherapy is a recommended treatment option for clinically localized prostate cancer. It involves using high energy rays or particles to kill cancer cells. External beam radiotherapy (EBRT) is the modern types of radiotherapy for prostate cancer. In EBRT, beams of radiation are focused on the prostate gland from a machine outside the body. The four-modern external beam radiation therapies for prostate cancer include three-dimensional conformal radiation therapy (3D-CRT), intensity modulated radiation therapy (IMRT), stereotactic body radiation therapy (SBRT) and proton beam radiation therapy (PBR). Radiation oncology is the most expensive treatment option for cancer [5, 6]. Several studies indicated that most treatment costs of cancer were dominated by direct medical costs of radiation therapy. Its total costs is also increased through time due to high demand [5–7]. Therefore, economic evaluation of such expensive treatment option is needed to minimize cost and to meet the current demand of the treatment.

The objective of this systematic review was to assess and identify studies that examined model-based economic evaluation of external beam radiation therapy for the treatment of localized prostate cancer.

## Methods

### Search strategy

A systematic review of the published literature was conducted in the period between November 2017 and January 2018 by the first author through PubMed, NHS EED (NHS Economic Evaluation Database, CRD York), and Cochrane databases using a specific search strategy (see Additional file 1: Appendix S1). The literatures were searched according to the preferred reporting items for systematic reviews and meta-analyses (PRISMA) statement [8]. At first, the author identified 1046 citations from all the databases. From PubMed 840 citations were identified using the term ‘cost–benefit analysis’, cost-effectiveness analysis’, ‘cost-utility analysis’ and ‘prostate cancer’. The similar search strategy was used for Cochrane and 79 citations were extracted. For NHS EED the term ‘prostate cancer’ was used to extract 127 citations. The extracted citations were imported into the Rayyan systematic review site [9] for inclusion or exclusion based on the defined criteria as described below. Studies included in this review were articles published between 2003 and 2017, and that conducted full-economic evaluations of the modality of external beam radiotherapy for the treatment of localized prostate cancer. Finally, 12 articles were selected for full review and included in the qualitative synthesis.

### Inclusion criteria

The target population includes men with prostate cancer, i.e., localized prostate cancer. Target studies focussed on full economic evaluations of prostate cancer (includes cost-effectiveness analysis, cost–benefit analysis, and cost-utility analysis). Modeling approaches based on decision trees, Markov cohort models, state-transition microsimulation models, and mathematical equations, in line with the definition of a “model” given by the “ISPOR Task Force on Good Research Practices—Modelling Studies” [10]. Only articles published in the English language is considered in this review. For the details of the inclusion criteria and patient intervention comparator outcome (PICO) strategy is presented in Additional file 1: Appendix S2.

### Exclusion criteria

Studies that are not full economic evaluations and studies that focus on diagnosis and screening of prostate cancer were excluded from the review. Abstracts were excluded if the source was a letter, editorial, review, commentary, methodological paper, abstracts without providing full information about the model and studies using models only as an illustration.

## Results

**Fig. 1:**
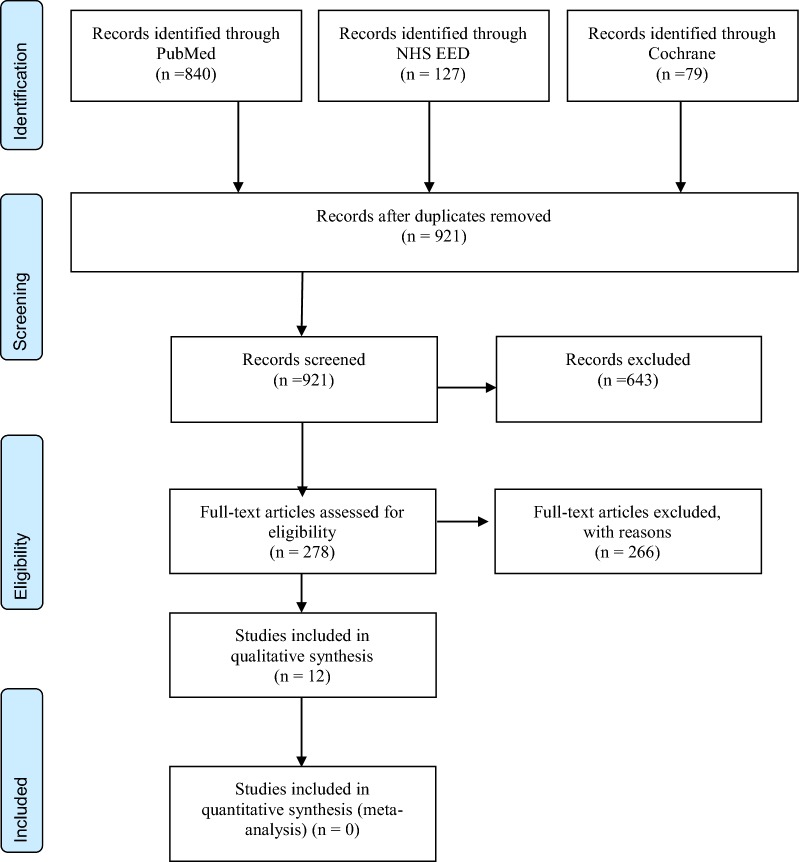
PRISMA 2009 flow diagram for model-based economic evaluations for localized prostate cancer [3]

Figure 1 shows the preferred reporting items for systematic reviews and meta-analyses diagram [8]. After duplicates were removed, 921 abstracts were screened. Of these, 643 articles were excluded and 278 full articles were assessed for eligibility.

### Summary of analytic framework, model features, methods and findings of included studies

Table 1 presents cost-effectiveness studies with full economic evaluations that compared IMRT with 3D-CRT for prostate cancer treatment. There are eight studies identified in this group and the year of the studies were from 2005 to 2016.

**Table 1:** Cost-effectiveness analyses comparing IMRT and 3D-CRT for prostate cancer

Study (authors, year)	Target population risk group	Time horizon/cycle length	Perspective/country	Data sources		Discount rate for costs and outcomes	Model type/type of evaluation/sensitivity analysis
				Effectiveness data	Cost data/year		
Carter et al. (2014) [11]	Hypothetical cohort of 65 years patients receiving radiotherapy to the prostate bed	20 years/1 year	3rd party payer perspective/Australia	Literature sources and their clinical interpretation, expert opinions	Literature sources, AR-DRG cost weights, medicare benefits schedule, pharmaceutical benefits schedule	Annual discount rate of 5% was used for costs and outcomes	Markov model/cost-utility analysis/one-way and probabilistic sensitivity analysis
Hummel et al. (2012) [12]	70-year-old-man with well-differentiated tumor	Lifetime/NA	Payer NHS/UK	Systematic literature review	Single-centre hospital costs, literature sources, expert opinion/2008	Costs and QALYs were discounted at a rate of 3.5% per year	Discrete event simulation model/cost-utility analysis/one-way and probabilistic sensitivity analysis
Konski et al. (2005) [13]	70-year-old man with a good and intermediate-risk prostate cancer	10 years	Payer US medicare	Administrative data: billing department at the Fox Chase Cancer Center	Medicare insurance		Markov model
Konski et al. (2006) [14]	70-year-old intermediate risk patients	15 years/1 year	3rd payer medicare perspective/US	Literature sources	Literature sources, drug red book/2005	Cost and benefits were discounted at 3% per year	Markov model/cost-utility analysis/probabilistic sensitivity analysis
Yong et al. (2012) [15]	Cohort of 70-year-old men with localized prostate cancer	Lifetime/1 month	Health system perspective/Canada	Literature sources	Activity-based costing, Ontario physician fee schedule, OCCI ambulatory care Ontario laboratory fee schedule, literature sources, drug formularies/2009	5% discount rate was used to adjust costs and quality-adjusted life-years	Markov model/cost-utility analysis/one-way sensitivity analysis
Cooperberg et al. (2013) [16]	Men aged 65 years old with low-risk, intermediate-risk, or high-risk prostate cancer	Lifetime	Payer US medicare	Literature review	Medicare cost data	Costs and QALY were discounted at 3% annually	Markov model
Zemplenyi et al. (2016) [17]	Low-, mid, and high-risk patients with localized prostate cancer	10 years/1 month	3rd party payer perspective/Hungary	Literature sources (three retrospective cohort studies and two RCTs)	Single-center cost collection study (micro-costing)	Costs and the quality-adjusted life years were discounted at a rate of 3.7% per year	Markov model cost-utility analysis/one-way and probabilistic sensitivity analysis, subgroup analysis

Most of the studies analyzed the cost effectiveness analysis for localized prostate cancer for patients aged 65 years old and above. The time horizon of the model for the studies were ranges from 10 years [13, 17], 15 years [14], 20 years [11] to lifetime [12, 15, 16]. Most of the papers report cost data from the payer’s perspective. There were no studies analyzed cost data from the hospital or societal perspective. All the studies were conducted in five different countries including four studies in US from the payer perspective [13, 14, 16]; Canada [15]; Australia [11]; UK [12] and Hungary [17].

For most of the studies the source of the effectiveness data is from published literature. The studies used a variety of sources for cost data including literature sources, AR-DRG cost weights, Medicare Benefits Schedule, Pharmaceutical Benefits Schedule, Medicare and Medicaid service was the main cost data sources for most of the US studies.

All the studies were full-economic evaluations using a Markov model [11, 13–17]. Carter et al. [11] estimated the cost effectiveness of IMRT compared to 3D-CRT using a Markov decision model by calculating the incremental quality adjusted life years (QALYs) and costs from the perspective of the Australian healthcare system. The study used a hypothetical cohort of 65 years old patients receiving radiotherapy to the prostate bed. A 20 years-time horizon with a 1-year cycle was used.

Using a discrete event simulation model, Hummel et al. [12] assessed cost-effectiveness from a UK National Health Service perspective.

Konski et al. [13] modelled a 70 year old population with a low and intermediate-risk prostate cancer. Cost analysis was performed from US Medicare payer’s perspective by taking cost data for men with Medicare insurance and prostate cancer treated with IMRT and 3D-CRT obtained from the billing department at the Fox Chase Cancer Center [13]. By another study Konski et al. [14] also discussed the cost-effectiveness of IMRT and 3D-CRT for a 70-year-old intermediate risk patients for a time horizon of 15 years using a Markov model. The model simulated four different health states including posttreatment, hormone therapy, chemotherapy, and death. The difference between the studies Konski et al. [13] and Konski et al. [14] is that the first study used a time horizon of 10 years and the latter use 15 years.

Yong et al. [15], the study used a clinical effectiveness estimates from a systematic review. At the base case analysis, the study assumed equal biochemical survival for IMRT and 3D-CRT, but lower frequency of gastrointestinal toxicity for IMRT. The cost data were estimated through activity-based costing, incorporating input from radiation oncologists, physicists and treatment planners from the Australian health care payer’s perspective [15]. The authors developed a life time Markov model with a cycle length of 1 month to compare the cost effectiveness of IMRT with 3D-CRT for men with clinically localized prostate cancer. The model includes six health states. The study conducted one-way sensitivity analyses to verify the logical consistency of the model and to assess the robustness of the model results by evaluating the cost-effectiveness of IMRT from different scenarios.

Cooperberg et al. [16] analyzed the cost effectiveness of different treatment options including radical prostatectomy and radiation therapy options for the treatment of three groups of patients risk patients i.e. men aged 65 years old with low-risk, intermediate-risk, or high-risk prostate cancer. The study used a lifetime Markov model to model a hypothetical man with low-, intermediate-, and high-risk prostate cancer over their lifetimes following primary treatment. The data for probabilities of outcomes were based on a systematic review. The study assumed that patients could experience remission, recurrence, salvage treatment, metastasis, death from prostate cancer, and death from other causes. Costs were determined from the US payer perspective, with incorporation of patient costs in a sensitivity analysis [16].

Zemplenyi et al. [17], the study used a Markov model with 10 years of time horizon and 1-month cycle length to calculate the incremental quality-adjusted life years and costs. The data for transition probabilities, adverse events and utilities were derived from relevant systematic reviews. The study used a cost data from a large university hospital and the cost analysis was done from the perspective of Hungarian third-party payer.

As shown in Table 2, there is one article comparing three external beam radiotherapy for prostate cancer [18]. Parthan et al. [18] analysed the cost effectiveness of three external beam radiation treatment modalities for the treatment of localized prostate cancer. These modalities were SBRT, IMRT and PBT. The study used a life time Markov cohort decision model for 65-year-old men with localized prostate cancer. The model incorporated the probabilities of experiencing treatment-related long-term toxicity or death with different possible disease states including all possible combinations of GI, genitourinary, sexual toxicity, and death. The study took toxicity probabilities from sources using meta-analytical techniques. The data for utilities and costs were taken from publicly available secondary sources (2011 Medicare reimbursements rates). The study calculated quality-adjusted life expectancy and expected lifetime cost per patient. The cost analysis was performed from both payer and societal perspectives by including lost work time during radiation treatment at 2011 wage rates estimated from the Bureau of Labor Statistics. One-way and probabilistic sensitivity analyses were per-formed.

**Table 2:** Cost-effectiveness analyses comparing IMRT, SBRT and PBT for localized prostate cancer treatment

Study (authors, year)	Target population risk group	Time horizon/cycle length	Interventions compared	Perspective/country	Data sources		Discount rate for costs and outcomes	Model type/type of evaluation/sensitivity analysis
					Effectiveness data	Cost data/year		
Parthan et al. (2012) [18]	65-year-old men with localized prostate cancer	Lifetime/not reported	SBRT vs. IMRT vs. PBT	3rd party Medicare payer and societal perspective/US	Published sources, meta-analytical techniques	Medicare rates, Bureau of Labour Statistic, literature sources, Red Book/2011	Costs and utilities discounted at 3.0% annually	Markov model/cost-utility analysis/one-way deterministic and probabilistic sensitivity analysis
Hodges et al. (2012) [19]	70-year-old low-to intermediate-risk patient with confined prostate cancer	10 years/1 year	IMRT vs SBRT	3rd party medicare payer perspective/US	Literature sources	Literature sources, medicare allowable costs, ambulatory payment classification/2010	Costs and utilities were discounted at a rate of 3% per year	Markov model/cost-utility analysis/probabilistic sensitivity analysis
Sher et al. (2014) [20]	65-year-old men with low-risk prostate cancer	Lifetime/4 months	IMRT vs. robotic and non-robotic SBRT	3rd party medicare payer perspective/US	Literature sources	Medicare payment schedule for hospital-based practice/2012		Markov model/cost-utility analysis/one-way deterministic and probabilistic sensitivity analysis
Lundkvist et al. (2005) [21]	Theoretical cohort age: 65-year	Lifetime	IMRT vs PBT	Societal Sweden	Literature sources	Literature sources	Costs and effects were discounted with 3% annually.	Markov model cost-utility analysis
Konski et al. (2007) [22]	Men aged 60 or 70 years old with intermediate-risk prostate cancer	NA	IMRT vs PBT	payer US medicare	literature	literature and from patient interviews	Costs and effects were discounted with 3% annually	Markov model

There are two articles comparing IMRT with SBRT [19, 20]. Hodges et al. [19] used a Markov decision model to simulate four disease states after radiation which could influence costs (no evidence of disease, hormone-responsive progression, hormone-refractory progression requiring chemotherapy, and death). The time horizon for the model was 10 years with 1-year cycles. The study took third party Medicare payer perspective using 2010 Medicare reimbursements rates. The study used effectiveness data from published literature. Sher et al. [20] modeled the cost-effectiveness of IMRT and robotic and non-robotic SBRT for 65-year-old men with low-risk prostate cancer. The authors constructed a lifetime Markov model with a life cycle of 4 months. The study was conducted in US from the third-party Medicare payer perspective using 2012 Medicare reimbursements. Disease, treatment, and toxicity data were extracted from the literature. The study performed both deterministic and probabilistic sensitivity analyses (PSA) were performed over a wide range of potential parameters.

On the other hand, there are two studies compared IMRT with PBT [21, 22]. Lundkvist et al. [21] assessed the cost-effectiveness of proton therapy in the treatment of four different cancers: left-sided breast cancer, prostate cancer, head and neck cancer, and childhood medulloblastoma. The study used a Markov cohort simulation model to simulate each cancer type and the life of patients treated with radiation. Cost and quality adjusted life years (QALYs) were used as primary outcome measures. The study calculated costs from a societal perspective in Sweden.

Konski et al. [22] used Markov model to incorporate health states of post-treatment, disease progression hormonally responsive (hormone therapy), disease progression hormonally unresponsive (chemotherapy), and death. The study assume that patients spend 1 year in each state before the opportunity to transition to another state or to stay in the same state.

### Main findings of economic evaluations identified in the systematic reviews

Table 3 shows the main findings of the cost effectiveness studies that compared IMRT with 3D-CRT.

**Table 3:** Main findings of Cost-effectiveness studies comparing IMRT and 3D-CRT for localized prostate cancer

Study (authors, year)	Results			Conclusions
	Mean cost	QALYs	ICER	
Carter et al. (2014) [11]	After 5 years IMRT: $9932 3D-CRT: $9706 After 20 years IMRT: $32,816 3D-CRT: $ 33,917	After 5 years IMRT: 4.244 3D-CRT: 4.239 After 20 years IMRT: 10.079 3D-CRT: 10.060	$41,572/QALY IMRT is dominant	IMRT was estimated to have a modest long-term advantage over 3D-CRT in terms of both improved effectiveness and reduced cost
Hummel et al. (2012) [12]	Scenario 1 IMRT: £6173 3D-CRT: £5184 Scenario 2 IMRT: £4946 3D-CRT: £4214 Scenario 3 IMRT: £4946 3D-CRT: £4486 Scenario 4 IMRT: £5687 3D-CRT: £7489	Scenario 1 IMRT: 6.802 3D-CRT: 6.792 Scenario 2 IMRT: 7.070 3D-CRT: 7.046 Scenario 3 IMRT: 7.070 3D-CRT: 6.983 Scenario 4 IMRT: 7.015 3D-CRT: 6.402	Scenario 1: £104,066/QALY Scenario 2: £31,162/QALY Scenario 3: £5295/QALY Scenario 4: dominant strategy	If IMRT can be used to prolong survival, it is very cost-effective. Otherwise, cost-effectiveness is uncertain
Konski et al. (2005) [13]	70 years old with intermediate risk IMRT: $33,837 3D-CRT: $21,377 70 years old with good risk IMRT: $31,950 3D-CRT: $19,213		70 years old with intermediate risk US$16,182/QALY 70 years old with good risk US$17,448/QALY	Intensity-modulated radiation therapy was found to be cost effective in the treatment of 70 years old man with prostate cancer
Konski et al. (2006) [14]	3D-CRT: $21,865 IMRT: $47,931	3D-CRT: 6.27 IMRT: 5.62	$40,101/QALY	IMRT therapy was found to be cost-effective at the upper limits of acceptability
Yong et al. (2012) [15]	3D-CRT $13,501 IMRT $14,520	3D-CRT: 6.062 IMRT: 6.085	$26,768/QALY	For radical radiation treatment of prostate cancer, IMRT seems to be cost-effective when compared with an equivalent dose of 3D-CRT
Cooperberg (2013) [16]	IMRT: $37,718 3D-CRT: $27,636	IMRT: 9.6 3D-CRT: 10.3	Not calculated	IMRT was found to be cost effective with a 0.5 QALYs gained
Zemplenyi et al. (2016) [17]	3D-CRT: €7160 IMRT: €6831 HF-IMRT: € 6.019	3D-CRT: €5.753 IMRT: €5.956 HF-IMRT: €5.957	3D-CRT dominated IMRT	Compared to 3D-CRT, both IMRT and HF-IMRT resulted in more health gains at a lower cost

Carter et al. [11] found that IMRT was dominant strategy i.e. both more effective and less costly than 3DCRT over 20 years. After 20 years the model estimated a cost for IMRT is $32,816 and for 3D-CRT it was $33,917. The estimated QALYs for the IMRT and 3D-CRT were 10.079 and 10.060 respectively. This showed an additional 20 QALYs gained and over $1.1 million saved per 1000 patients treated over 20 years. The authors performed a one-way sensitivity analysis and showed that the model was highly robust to changes in individual parameters and IMRT remained the dominant treatment in all scenarios. For the probabilistic sensitivity analysis, the authors also showed that IMRT had an 86% probability of being dominant and a 93% probability of being a cost-effective treatment given an ICER threshold of $50,000 per QALY.

On the other study Hummel et al. [12] the authors used different scenarios to analyses the cost effectiveness of IMRT vs 3DCRT strategies. In scenarios where, estimated survival was greater for IMRT than 3DCRT, IMRT was clearly cost-effective (ICER < £20,000). For scenarios where only, a difference in late gastrointestinal (GI) toxicity was assumed, the ICER was highly sensitive to uncertain model parameters, including the magnitude of the difference, the duration of gastrointestinal toxicity and the cost difference between treatments. For the most likely scenario, a 15% difference in late gastrointestinal toxicity, the ICER was £35,000, with a 20% probability that it is cost-effective at a maximum threshold of £20,000 and a 48% probability at a threshold of £30,000 [12]. The authors performed a univariate sensitivity analyses on key parameters, such as age, incremental cost of IMRT in comparison with 3D-CRT, and duration of late GI toxicity. Finally, the authors concluded that IMRT was used to prolong survival and it is cost-effective.

In the findings of Konski et al. [13], IMRT was also found to be cost effective in the treatment of a 70 year old man with prostate cancer. The incremental cost-effectiveness ratio of US$16,182/QALY for men with intermediate-risk prostate cancer and US$17,448/QALYs for men with good-risk prostate cancer. The authors also performed a sensitivity analysis and found that a longer time horizon and younger age affected the cost-effectiveness ratio. A similar study by Konski et al. [14] found that the mean cost of IMRT and 3D-CRT were $47,931 and $21,865 respectively. The estimated amount of QALY for IMRT and 3D-CRT were 6.27 and 5.62. The incremental cost-effectiveness comparing IMRT with 3D-CRT was $40,101/QALYs. Cost-effectiveness acceptability curve analysis revealed a 55.1% probability of IMRT being cost-effective at a $50,000/QALY willingness to pay. Konski et al. [14] concluded that IMRT was found to be cost-effective at this upper limits cost effectiveness acceptability curve. The results, however are dependent on the assumptions of improved biochemical disease-free survival with fewer patients undergoing subsequent salvage therapy and improved quality of life after the treatment. The authors suggested that in the absence of prospective randomized trials, decision analysis can help inform physicians and health policy experts on the cost-effectiveness of emerging technologies [14].

The result of a study by Yong et al. [15] found that the mean cost for IMRT and 3D-CRT was $14,520 and $13,501 with a QALY 6.085 and 6.062 respectively. IMRT produced 0.023 more QALY than 3D-CRT at an additional cost of $621 (QALY and costs discounted at 5% per year), yielding an incremental cost-effectiveness ratio of $26,768 per QALY gained. The treatment cost of IMRT was $1019 more than 3D-CRT, but IMRT resulted in less frequent gastrointestinal toxicity, thus avoiding $402 in the treatment of toxicity. In the scenario that compared a higher dose of IMRT (75.6 Gy) to 3D-CRT (68.4 Gy), IMRT improved disease control with equal toxicity incidence, and the IMRT strategy dominated (less costly and more effective). In the base case scenario (no survival difference), the cost-effectiveness of IMRT was most sensitive to the treatment cost difference between IMRT and 3DCRT.

Cooperberg [16] studied the cost effectiveness of different surgical techniques and radiotherapy options including dose-escalated 3D-CRT, IMRT, brachytherapy, or combination using a life time Markov model for hypothetical men with low-, intermediate-, and high-risk prostate cancer. In the case for patients with low risk prostate cancer the study found that the mean life time cost for IMRT was $37,718 and for 3D-CRT $27,636. The associated QALY for IMRT and 3D-CRT were 9.6 and 10.3. Therefore, in this study IMRT was found to be cost effective for patients with low risk prostate cancer.

Zemplenyi et al. [17] compared the cost-effectiveness of high-dose IMRT and hypo fractionated IMRT versus conventional dose 3D-CRT for the treatment of localised prostate cancer. The study found the expected mean lifetime cost for 3DCRT, IMRT and hypo fractionated IMRT were 7160 euros, 6831 euros and 6019 euros respectively. The expected quality-adjusted life years (QALYs) were 5.753 for 3DCRT, 5.956 for IMRT and 5.957 for HFIMRT. The study concluded that compared to 3DCRT, both IMRT and hypo fractionated IMRT resulted in more health gains at a lower cost.

Table 4 presents the main findings for cost-effectiveness studies of IMRT, SBRT and PBT for localized prostate cancer. The cost effectiveness analysis result by Hodges et al. [19] revealed that the mean cost of SBRT was US$22,152 with 7.9 QALYs. However, the mean cost for IMRT was US$35,431 and 7.9 QALYs, respectively. ICER was not calculated for this analysis because of the QALYs were the same for both SBRT and IMRT. Based on this result, compared with IMRT, SBRT was considered cost-effective. The authors performed a sensitivity analysis and the sensitivity analysis indicated that if the SBRT cohort experienced a decrease in quality of life of 4% or a decrease in efficacy of 6%, then SBRT would no longer dominate IMRT in cost-effectiveness [19]. This sensitivity analysis also indicated that the ICER for SBRT over IMRT was less than $50,000/QALY in 66% of the sensitivity analysis scenarios.

**Table 4:** Main findings of Cost-effectiveness studies comparing IMRT, SBRT and PBT for localized prostate cancer

Study (authors, year)	Result			Conclusions
	Mean cost	QALYs	ICER	
Hodges et al. (2012) [19]	SBRT: $22,152 IMRT: $35,431	SBRT = 7.9 IMRT = 7.9	NA	“Compared with IMRT, SBRT for low- to intermediate-risk prostate cancer has great potential cost savings”
Sher et al. (2014) [20]	IMRT: $27,564 Non-robotic SBRT: $10,108 Cyber-knife SBRT: $19,275	IMRT: 9.96 SBRT: 9.93	IMRT vs robotic SBRT: $285,000/QALY IMRT vs Non- robotic: US$591,100/QALY	“SBRT clearly contained more value than IMRT for external-beam treatment”
Parthan et al. (2012) [18]	Payer perspective IMRT: $33,068 PBT: $69,412 SBRT: $24,873 Societal perspective SBRT: $25,097, IMRT: $35,088 PBT: $71,657	SBRT: 8.11 IMRT: 8.05 PBT: 8.06	SBRT was dominating over RT and IMRT (less costly and more QALYs)	“Based on the assumption that each treatment modality results in equivalent long-term efficacy, SBRT is a cost-effective strategy resulting in improved quality-adjusted survival compared to IMRT and PT for the treatment of localized prostate cancer”
Lundkvist, et al. (2005) [21]	Proton: €13,491 IMRT: €5477	Cost Standard case result: 7952.6 High proton radiation cost estimate: 10,485.2 Low proton radiation cost estimate: 7343.9	QALY Standard case result: 0.297 High proton radiation cost estimate: 0.297 Low proton radiation cost estimate: 0.297	“Investment in a proton facility may thus be cost-effective. The results must be interpreted with caution, since there is a lack of data, and consequently large uncertainties in the assumptions used”
Konski et al. (2007) [22]	70-year-old man PBT: $63,511 IMRT: $36,808 60-year-old man PBT: $64,989 IMRT: $39,355	70-year-old man PBT: 8.54 IMRT: 8.12 60-year-old man PBT: 9.91 IMRT: 9.45	70-year-old man $63,578/QALY 60-year-old man $55,726/QALY	“Even when based on the unproven assumption that protons will permit a 10-Gy escalation of prostate dose compared with IMRT photons, proton beam therapy is not cost effective for most patients with prostate cancer using the commonly accepted standard of $50,000/QALY”

On the other hand, the cost effectiveness analysis by Sher et al. [20] found that the cost of IMRT, non-robotic SBRTand Cyber-knife SBRT were $27,564, $10,108 and $19,275 respectivelly. The QALY for IMRT (9.96) was slightly higher than after SBRT (9.93) under the assumption of worse toxicity after SBRT [20]. The ICER for IMRT over robotic SBRT and non-robotic SBRT were US$285,000 and US$591,100/QALY, respectively. The authors concluded that SBRT clearly contained more value than IMRT for external-beam treatment. After the sensitivity analysis SBRT was almost always the cost-effective therapy, in which the ICER for IMRT was generally over $100,000/QALY. Reimbursement for Robotic-SBRT versus non-robotic -SBRT significantly influenced its ICER. Treatment efficacy, rectal toxicity and impotence, and the potential for unforeseen SBRT late effects were the most critical parameters in the model; when including these uncertain parameters in a PSA, SBRT was still most likely to be cost-effective at a willingness to pay of $100,000/QALY.

Parthan et al. [18] compared three external beam radiation therapy alternatives and their result showed that compared to IMRT and PBT, SBRT was less costly and resulted in more QALYs. The authors conducted their analysis both from the payer’s and societal perspective. From the payer’s perspective the life time cost of SBRT, IMRT and PBT were $24,873, $33,068 and $69,412 respectively. Whereas, from the societal perspective where the lost work time was calculated, the life time costs were $25,097, $35,088 and $71,657 respectively. In both perspectives the authors found that SBRT was the least expensive. The QALYs for SBRT, IMRT and PBT were 8.11, 8.06, and 8.05 respectively. The authors performed a sensitivity analysis and the conclusions in the base-case scenario were robust with respect to variations in toxicity and cost parameters consistent with available evidence [18]. The authors showed that from the payer’s perspective, at a threshold of $50,000/QALY, SBRT was cost-effective in 75% and 94% of probabilistic simulations compared to IMRT and PT, respectively. From a societal perspective, SBRT was cost-effective in 75% and 96% of simulations compared to IMRT and PT, respectively, at a threshold of $50,000/QALY. In threshold analyses, SBRT was less expensive with better outcomes compared to IMRT at toxicity rates 23% greater than the SBRT base-case rates. In general, the authors concluded that SBRT was cost-effective, resulting in cost savings and improved QALYs compared to IMRT and PBT for the treatment of localized prostate cancer. From the above studies that compared IMRT, SBRT and PBT in all studies SBRT was found to be cost effective.

Lundkvist et al. [21] assessed the cost-effectiveness of proton therapy compared with IMRT for different cancer types including left-sided breast cancer, prostate cancer, head and neck cancer, and childhood medulloblastoma. The study assumed that 300 prostate cancer patients per year would be treated with proton therapy. The study found that the total radiation cost for prostate cancer was €5477 and the cost of proton was estimated €13,491. The average cost per QALY gained for the standard case was about €7952.6 with the total numbers of gained QALYs were calculated to 0.30. The study concluded that investment in proton facility is cost-effective. However, the cost effectiveness of proton facility depends on the total cancer patients treated since the authors studied several types of cancer.

The analysis by Konski et al. [22] found that the expected mean cost of for a time horizon of 15 years for proton beam therapy and IMRT of $63,511 and $36,808, and $64,989 and $39,355 for a 70-year-old and 60-year-old man respectively, with quality-adjusted survival of 8.54 and 8.12 and 9.91 and 9.45 QALY, respectively. The incremental cost effectiveness ratio was calculated to be $63,578/QALY for a 70-year-old man and $55,726/QALY for a 60-year-old man. The authors performed sensitivity analysis and showed that the probability of cost effectiveness increases as the willingness to pay increases. The probability of cost effectiveness is 49% at a willingness to pay of $50,000/quality-adjusted life-year.

The critical appraisal of the included studies according to Drummond’s 10 item check list is illustrated in Additional file 1: Appendix S3. From the included 12 economic evaluations, 10 studies scored ≥ 9 points. Two studies [16, 21] scored 5 points. The items that most frequently failed was about effectiveness. Out of 12 studies, 8 studies do not satisfy the conditions for program effectiveness.

## Discussion and conclusion

In this systematic review, 12 studies were identified that investigated the cost effectiveness of different external beam radiation therapy options for the treatment of localized prostate cancer. Mainly there were four treatment modalities included in the review. These are three-dimensional conformal radiation therapy (3D-CRT), intensity modulated radiation therapy (IMRT), stereotactic body radiation therapy (SBRT) and proton beam radiation therapy (PBR). Articles published between 2003 and 2017 that conducted full-economic evaluations were included in the review. From 12 articles 7 studies were compared the cost effectiveness of IMRT with 3D-CRT [11–17]), one article compared three external beam radiation therapy of IMRT, SBRT and PBT [18] and two article compared IMRT with SBRT [19, 20]. The other two articles [21, 22] were compared the cost effectiveness of IMRT with PBT for the treatment of prostate cancer.

For most of the studies the target population of the analysis were those men with prostate cancer aged above 65 years and 70 years. This shows that Prostate cancer affect most of the older population. Most of the studies were conducted from the US and from the health care payer perspective. The result of most of the studies indicated that IMRT was found to be cost-effective when it is compared with 3D-CRT. Cartel et al. [11] showed that IMRT was a dominant strategy. On the other way SBRT was found to be cost effective when compared with IMRT.

There are limited number of studies exist on the cost effectiveness of radiation therapy options for the treatment of localize prostate cancer across Europe. Most studies are originated from the US Medicare payer Perspective. Further research is need that investigate the cost effectiveness of these radiation therapy options from the societal perspective in Europe. Evidence base cost effectiveness studies based on market demand for medical services remain significant for future studies especially in larger society settings.

## Additional file

**Additional file 1: Appendix S1.** Search strings used for systematic search of economic evaluations of prostate cancer. **Appendix S2**. Patient Intervention Comparator Outcome (PICO) strategy. **Appendix S3**. Critical Appraisal of the selected published literatures for systematic review.

## Data Availability

All data generated or analysed during this study are included in this published article and its Additional file.

## Abbreviations

2-DRT: two-dimensional radiotherapy
3D-CRT: three-dimensional conformal radiotherapy
ICER: incremental cost effectiveness ratio
IMRT: intensity-modulated radiation therapy
EBRT: external beam radiotherapy
GI: gastrointestinal
ISPOR: International Society for Pharmacoeconomics and Outcomes Research ISPOR
LE: life expectancy
NHS EED: National Health System Economic Evaluation Database
PBT: proton beam therapy
PRISMA: preferred reporting items for systematic reviews and meta-analyses
QALYs: quality-adjusted life years
RCT: randomized control trial
SBRT: stereotactic body radiation therapy
UK: United Kingdom
US: United States

## References

[1] Drummond MF, Sculpher MJ, Torrance GW, O’Brien BJ, Stoddart GL (2005). Methods for the economic evaluation of health care programme.

[2] American Cancer Society (2018). Facts & figures 2018.

[3] Bray F, Kiemeney LA, Bolla M, van Poppel H (2017). Epidemiology of prostate cancer in Europe: patterns, trends and determinants. Management of prostate cancer: a multidisciplinary approach.

[4] National Collaborating Centre for Cancer (2014). Prostate cancer: diagnosis and treatment.

[5] Jakovljevic M, Zugic A, Rankovic A, Dagovic A (2015). Radiation therapy remains the key cost driver of oncology inpatient treatment. J Med Econ.

[6] Dagovic A, Walstra KM, Gutzwiller FS, Djordjevic N, Rankovic A, Djordjevic G (2014). Resource use and costs of newly diagnosed cancer initial medical care. Eur J Oncol.

[7] Jakovljević M, Ranković A, Rančić N, Jovanović M, Ivanović M, Gajović O (2013). Radiology services costs and utilization patterns estimates in Southeastern Europe—a retrospective analysis from Serbia. Value Health Reg Issues.

[8] Moher D, Liberati A, Tetzlaff J, Altman DG, Prisma Group (2009). Preferred reporting items for systematic reviews and meta-analyses: the PRISMA statement. PLoS Med..

[9] Ouzzani M, Hammady H, Fedorowicz Z, Elmagarmid A (2016). Rayyan—a web and mobile app for systematic reviews. Syst Rev.

[10] Caro JJ, Briggs AH, Siebert U, Kuntz KM (2012). Modeling good research practices–overview: a report of the ISPOR-SMDM modeling good research practices task force-1. Med Decis Mak.

[11] Carter HE, Martin A, Schofield D, Duchesne G, Haworth A, Hornby C (2014). A decision model to estimate the cost-effectiveness of intensity modulated radiation therapy (IMRT) compared to three dimensional conformal radiation therapy (3DCRT) in patients receiving radiotherapy to the prostate bed. Radiother Oncol.

[12] Hummel SR, Stevenson MD, Simpson EL, Staffurth J (2012). A model of the cost-effectiveness of intensity-modulated radiotherapy in comparison with three-dimensional conformal radiotherapy for the treatment of localised prostate cancer. Clin Oncol.

[13] Konski A (2005). Cost-effectiveness of intensity-modulated radiation therapy. Expert Rev Pharmacoecon Outcomes Res.

[14] Konski A, Watkins-Bruner D, Feigenberg S, Hanlon A, Kulkarni S, Beck JR (2006). Using decision analysis to determine the cost-effectiveness of intensity-modulated radiation therapy in the treatment of intermediate risk prostate cancer. Int J Radiat Oncol Biol Phys.

[15] Yong JH, Beca J, McGowan T, Bremner KE, Warde P, Hoch JS (2012). Cost-effectiveness of intensity-modulated radiotherapy in prostate cancer. Clin Oncol R Coll Radiol.

[16] Cooperberg MR, Ramakrishna NR, Duff SB, Hughes KE, Sadownik S, Smith JA (2013). Primary treatments for clinically localised prostate cancer: a comprehensive lifetime cost-utility analysis. BJU Int.

[17] Zemplenyi AT, Kalo Z, Kovacs G, Farkas R, Beothe T, Banyai D (2016). Cost-effectiveness analysis of intensity-modulated radiation therapy with normal and hypofractionated schemes for the treatment of localised prostate cancer. Eur J Cancer Care Engl.

[18] Parthan A, Pruttivarasin N, Davies D, Taylor DC, Pawar V, Bijlani A (2012). Comparative cost-effectiveness of stereotactic body radiation therapy versus intensity-modulated and proton radiation therapy for localized prostate cancer. Front Oncol.

[19] Hodges JC, Lotan Y, Boike TP, Benton R, Barrier A, Timmerman RD (2012). Cost-effectiveness analysis of stereotactic body radiation therapy versus intensity-modulated radiation therapy: an emerging initial radiation treatment option for organ-confined prostate cancer. J Oncol Pract.

[20] Sher DJ, Parikh RB, Mays-Jackson S, Punglia RS (2014). Cost-effectiveness analysis of SBRT versus IMRT for low-risk prostate cancer. Am J Clin Oncol.

[21] Lundkvist J, Ekman M, Ericsson SR, Jonsson B, Glimelius B (2005). Proton therapy of cancer: potential clinical advantages and cost-effectiveness. Acta Oncol.

[22] Konski A, Speier W, Hanlon A, Beck JR, Pollack A (2007). Is proton beam therapy cost effective in the treatment of adenocarcinoma of the prostate?. J Clin Oncol.

