# Supplementary material for: Model-based cost-effectiveness analysis of external beam radiation therapy for the treatment of localized prostate cancer: a systematic review
Source: Cost Eff Resour Alloc. 2019 May 21;17:10. doi: 10.1186/s12962-019-0178-3 (PMC6528358; doi:10.1186/s12962-019-0178-3)
Supplement: Supplementary file 1 — Additional file 1: Appendix S1. Search strings used for systematic search of economic evaluations of prostate cancer. Appendix S2. Patient Intervention Comparator Outcome (PICO) strategy. Appendix S3. Critical Appraisal of the selected published literatures for systematic review. [file 12962_2019_178_MOESM1_ESM.docx]

**Additional Materials**

**Appendix S1**: Search strings used for systematic search of economic evaluations of prostate cancer

| **Database** | **Year** | **Search strategy** | **Reference identified** |
| --- | --- | --- | --- |
| **NHS EED** | 2017 | ((Prostate cancer)) and ((Economic evaluation: ZDT and Bibliographic: ZPS) OR (Economic evaluation: ZDT and Abstract: ZPS)) IN NHSEED | 127 |
| **PubMed** | 2017 | ((Cost-Benefit Analysis [Mesh] OR “cost-benefit analysis” OR “cost-utility analysis” OR “cost-effectiveness analysis”) AND ("Prostate cancer"[Mesh] OR "Prostate cancer")) | 840 |
| **Cochrane**  **library** | 2017 | ("Cost-Benefit Analysis" or "cost-utility analysis" or "cost-effectiveness analysis") and ("Prostate cancer" or "prostate cancer treatment") in Title, Abstract, Keywords in Economic Evaluations' | 79 |
| **Total number of citations identified** | | | 1,046 |

**Appendix S2:** Patient Intervention Comparator Outcome (PICO) strategy.

| **Criteria** |  |
| --- | --- |
| Population | Men with localized prostate cancer |
| Intervention | Treatments for localized prostate cancer:  External Beam Radiation Therapy |
| Comparators | Three-Dimensional Conformal Radiation Therapy (3D-CRT)  Intensity Modulated Radiation Therapy (IMRT)  Stereotactic Body Radiation Therapy (SBRT)  Proton Beam Radiation Therapy (PBRT) |
| Outcomes | Incremental cost per quality-adjusted life-year (primary outcome) Incremental cost-effectiveness ratios (ICERs)  Cost-effectiveness Analysis  Cost-Benefit Analysis  Cost-Utility Analysis |
| Timepoints/ follow-up | Any |
| Study type | Cost-effectiveness, cost-benefit or cost-utility studies  Comparative studies |
| Publication date | January 2003– December 2017 |
| Publication language | English |
| Setting | Any country |

**Appendix S3:** Critical Appraisal of the selected published literatures for systematic review

The quality assessment of the systematic reviews was made based on Drummond’s Checklist for all the studies. Accordingly, from 12 economic evaluation studies 10 studies were scored a point 9 and above.

| **Drummond’s Checklist questions (yea/no/Can’t tell**) | Carter et al  (2014) | Hummel et al  (2012) | Konski et al  (2005) | Konski et al  (2006) | Yong et al  (2012) | Cooperberg  (2013) | Zemplenyi et al  (2016) | Hodges et al  (2012) | Sher et al  (2014) | Parthan et al  (2012) | Lundkvist, et al.  (2005) | Konski et al  (2007) |
| --- | --- | --- | --- | --- | --- | --- | --- | --- | --- | --- | --- | --- |
| 1. Was a well-defined question posed in answerable form? | Yes | Yes | Yes | Yes | Yes | Yes | Yes | Yes | Yes | Yes | Yes | Yes |
| 2. Was a comprehensive description of the competing alternatives given? | Yes | Yes | Yes | Yes | Yes | No | Yes | Yes | Yes | Yes | Yes | Yes |
| 3. Was there evidence that the programmers’ effectiveness had been established? | No | Yes | Yes | Yes | No | No | Yes | Can’t Tell | Can’t Tell | No | No | No |
| 4. Were all important and relevant costs and consequences for each alternative identified? | Yes | Yes | Yes | Yes | Yes | Can’t Tell | Yes | Yes | Yes | Yes | No | Yes |
| 5. Were costs and consequences measured accurately in appropriate physical units? | Yes | Yes | Yes | Yes | Yes | Yes | Yes | Yes | Yes | Yes | No | Yes |
| 6. Were costs and consequences valued credibly? | Yes | Yes | Yes | Yes | Yes | Yes | Yes | Yes | Yes | Yes | Can’t Tell | Yes |
| 7. Were costs and consequences adjusted for differential timing? | Yes | Yes | Yes | Yes | Yes | Yes | Yes | Yes | Yes | Yes | Yes | Yes |
| 8. Was an incremental analysis of costs and consequences of alternatives performed? | Yes | Yes | Yes | Yes | Yes | No | Yes | Yes | Yes | Yes | Yes | Yes |
| 9. Was allowance made for uncertainty in the estimates of costs and consequences? | Yes | Yes | Yes | Yes | Yes | Yes | Yes | Yes | Yes | Yes | Yes | Yes |
| 10. Did the presentation and discussion of the study results include all issues of concern to users? | Yes | Yes | Yes | Yes | Yes | Can’t Tell | Yes | Yes | Yes | Yes | Can’t Tell | Yes |
| **Score** | 9 | 10 | 10 | 10 | 9 | 5 | 10 | 9 | 9 | 9 | 5 | 9 |
